# The treatment of PTSD in an older adult Norwegian woman using narrative exposure therapy: a case report

**DOI:** 10.1080/20008198.2017.1414561

**Published:** 2018-01-16

**Authors:** Nina Mørkved, Steven R. Thorp

**Affiliations:** ^a^ Mosjøen District Psychiatric Centre, Helgeland Hospital, Mosjøen, Norway; ^b^ Department of Psychology, University of Tromsø, Tromsø, Norway; ^c^ California School of Professional Psychology, Alliant International University, San Diego, CA, USA

**Keywords:** Psychotherapy, trauma narratives, sexual abuse, emotional abuse, complex trauma, psicoterapia, narrativas de trauma, abuso sexual, abuso emocional, trauma complejo, 心理治疗, 创伤叙述, 性虐待, 情绪虐待, 复杂创伤, • PTSD due to single or multiple traumatic events can occur at any age and persist over years.• The majority of research on PTSD treatments is focused on younger and middle-aged adults.• There is a need for research on how these treatment-protocols might be generalizable to older adults.• This case report illustrates that narrative exposure therapy was effective in treating PTSD and depressive symptoms in an older adult.

## Abstract

The bulk of the literature on effective treatments for posttraumatic stress disorder (PTSD) has focused on children, adolescents, and young adults. The evidence on treatments for older adults is sparse. This case report presents the application of narrative exposure therapy (NET) for a 70-year-old Norwegian woman suffering from PTSD as a result of multiple childhood and late life traumatic events. NET is a manualized, short-term, cognitive-behavioural therapy for PTSD, originally developed to meet the needs of survivors of war and organized violence. Some aspects of NET may be a good fit for older adults, including its brevity, simplicity, and concrete nature. The course of therapy included psychoeducation, a lifeline exercise, imaginal exposure, and the creation of a coherent narrative. Symptoms of depression and posttraumatic stress showed improvement over the course of therapy and at follow-up. This suggests that NET may have potential as a trauma treatment for older adults.

## Abstract (es)

La mayor parte de la literatura sobre tratamientos efectivos para el trastorno de estrés postraumático (TEPT) se ha centrado en niños, adolescentes y adultos jóvenes. La evidencia sobre tratamientos para adultos mayores es escasa. Este informe de caso presenta la aplicación de la terapia de exposición narrativa (NET, por sus siglas en inglés) para una mujer noruega de 70 años que padece TEPT como resultado de múltiples acontecimientos traumáticos en la infancia y en los últimos años. NET es una terapia cognitivo-conductual manualizada y de corto plazo para el TEPT, desarrollada inicialmente para satisfacer las necesidades de los supervivientes de guerras y de violencia organizada. Algunos aspectos de la NET pueden ser adecuados para adultos mayores, incluyendo su brevedad, su simplicidad y su naturaleza concreta. El curso de la terapia incluyó psicoeducación, un ejercicio de línea de vida, exposición imaginal y la creación de una narrativa coherente. Los síntomas de depresión y estrés postraumático mostraron una mejoría en el curso de la terapia y en el seguimiento. Esto sugiere que NET puede tener potencial como tratamiento de trauma para personas mayores.

## Abstract (zh)

大量文献对创伤后应激障碍（PTSD）的有效治疗集中在儿童、青少年和年轻成人。关于老年的治疗证据非常少见。这个个案报告呈现了应用叙事暴露疗法（NET）对一个70岁的因多种童年创伤和晚年生活创伤事件而患有 PTSD的挪威女性进行治疗。NET 是一个手册化的、短程的、针对PTSD 的认知行为疗法。最初是为了治疗战争幸存者和机构暴力而开发的。NET 的某些方面（包括它的简洁、简单和具体）可能适合更年老的成人。治疗的过程包括心理教育、生命线练习，想象暴露和创作连贯叙事。抑郁和创伤后应激症状在治疗过程以及治疗后得到改善。这说明 NET 可能有治疗年老成人创伤的潜力。

## 1. Introduction

Older adults comprise an increasing proportion of the population in industrialized countries (Böttche, Kuwert, & Knaevelsrud, 2012). A recent study reports a 4.5% lifetime prevalence of posttraumatic stress disorder (PTSD) in older adults, with higher rates for women as compared to men (Pietrzak, Goldstein, Southwick, & Grant, 2012). Research on effective treatments for older patients suffering from PTSD is relatively sparse (Böttche et al., 2012). The majority of research on effective treatments for PTSD has focused on children, adolescents, and younger and middle-aged adults, and there are few representative epidemiological studies on the consequences of multiple traumas in older adults (Clapp & Beck, 2012).

There is a need for research on how protocols for treating PTSD in younger and middle-aged adults might be generalizable for older adults. There is one case report illustrating the efficacy of imaginal exposure in treating PTSD in a 57-year-old woman with a history of childhood sexual abuse (Russo, Hersen, & Van Hasselt, 2001). Thorp, Glassman, and Wells (2015) noted that, while there have been small published studies showing the promise of psychotherapies for older adults with PTSD (e.g. a small clinical trial describing an exposure-based intervention by Thorp, Stein, Jeste, Patterson, & Wetherell, 2012), it is not yet clear whether standard protocols for the general population will suffice for older adults. Thorp et al. (2015) reviewed some modifications that have been proposed for standard treatments, including increasing the structure of treatment, progressing at a slower pace, utilizing memory aids, and simplifying materials. A review by Böttche et al. (2012) indicates that disorder-specific interventions combined with narrative life-review approaches may be effective.

Narrative exposure therapy (NET) is a manualized, short-term cognitive behavioural therapy (CBT) for PTSD that utilizes aspects of life review techniques (Schauer, Neuner, & Elbert, 2011). The majority of research has focused on the effectiveness of NET in the treatment of refugees and asylum seekers with PTSD, and it has demonstrated a significant reduction of PTSD symptoms and low dropout rates (see Mørkved et al., 2014, for review). NET has an explicit focus on imaginal exposure to traumatic events, with narration of a coherent full autobiography (Schauer et al., 2011).

After the NET therapist provides psychoeducation on PTSD, the client and the therapist create a chronological physical lifeline that includes all significant memories (including traumatic, unpleasant, and happy ones) from birth to present. Thus, in contrast to the treatments with the strongest support for PTSD, prolonged exposure therapy (Foa, Hembree, & Rothbaum, 2007) and cognitive processing therapy (Resick & Schnicke, 1993), NET encompasses all major life events, is simpler and briefer, and it utilizes physical objects as memory aids (Mørkved et al., 2014). NET also includes a written account of the traumatic event, produced and read by the therapist during sessions, which could also be beneficial for older adults. Unlike prolonged exposure therapy, NET also does not include an in vivo (situational) exposure component, which could make the treatment more efficient and more palatable to some older adults if outcomes are comparable.

There is a paucity of research on NET in older adults with PTSD as a result of childhood and later life traumas. Bichescu et al., (2007) investigated NET for older adults suffering from PTSD. Nine male Romanian older adults with PTSD as a result of political violence completed five sessions of NET, and had better outcomes compared to the comparison group receiving psychoeducation only. Some researchers have suggested that NET is well suited to PTSD involving multiple traumatic events (Mørkved et al., 2014). The aim of the current case study is to highlight how NET was a feasible and effective trauma treatment for an older Caucasian woman suffering from PTSD as a consequence of childhood and lifetime traumas.

## 2. Case introduction

A 70-year-old married Caucasian Norwegian woman, for whom we use the pseudonym Lotte, was referred to outpatient psychological treatment after being diagnosed with PTSD and Recurring Depressive Episodes according to the *International Classification of Mental Disorders – 10* (WHO, 1992). The client provided informed written consent for this case report – which was not conducted as part of any larger study. The study was approved by the Regional Committees for Medical and Health Research Ethics North (2015–1980) and the Norwegian Centre for Research Data. We omitted and altered non-crucial information to ensure anonymity.

### 2.1. Presenting complaints

At intake, Lotte presented with posttraumatic symptoms: hyperarousal, anxiety, bodily dissociation, severe nightmares, disturbed sleep and fatigue, and decreased social functioning. She described avoidance of both internal triggers (e.g. thoughts, memories) and external triggers (e.g. sounds, places) related to traumatic experiences. She reported feeling depressed, and expressed reduced hope for the future. In addition, Lotte expressed dysfunctional assumptions about her value as a human being and difficulties regulating her emotions. She was not suicidal at intake or during the course of treatment.

### 2.2. Case history

Lotte had survived experienced emotional abuse and emotional neglect starting at age 3, childhood sexual abuse from age 6 until age 14, and multiple traumatic experiences in adolescence and adulthood. At 15 years old, her mother blamed her brother’s death on Lotte because she lent her brother money for the bus fare to go skiing and he was killed in an avalanche. Later, when Lotte’s boyfriend determined that she was not a virgin, he left her. At age 18, Lotte reported feeling shameful and overdosed on sleeping pills. Lotte’s mother found her in time to take her to the hospital, where she was revived.

Lotte married at 21, and had two daughters. When she was 40 years old, she discovered that her husband was having an affair which led to a divorce. Three years later, Lotte was shocked when she learned that her ex-husband had died in a shipwreck. At the age of 50, an accident severed Lotte’s upper spine. This forced her retire from work earlier than planned.

At age 65, Lotte’s health began deteriorating. She started to experience intrusive memories, symptoms of anxiety and depressed mood, nightmares, headaches, increased pain in her back and neck, and social isolation. She saw a neurologist for an assessment of the pain in her neck and spine. As he inquired about her past, she told him about her sexual abuse and other traumatic events. She was referred for a psychiatric assessment and stabilizing inpatient treatment, where she was diagnosed with PTSD. She received psychoeducation about PTSD symptoms, triggers, mindfulness, and grounding techniques. However, the PTSD and depressive symptoms continued, so she was referred to outpatient trauma-focused psychotherapy that resulted in the NET trial described in this report. She was prescribed antidepressants for decades (and during this trial) before seeking psychotherapy, but she had clinically significant PTSD symptoms at the start of this study (indicating that the medication was insufficient for reducing PTSD severity) and her dosage was not adjusted during the course of NET.

## 3. Method

### 3.1. Assessment

The symptoms of PTSD, depression, and anxiety were measured using the Norwegian versions of the following instruments:

The Posttraumatic Symptom Scale Self-Report (PSS-SR; Foa, Riggs, Dancu, & Rothbaum, 1993) is a 17-item self-report questionnaire designed to assess the presence and severity of PTSD symptoms in traumatized individuals (Foa et al., 1993). The PSS-SR has shown good sensitivity, satisfactory internal consistency, high test-retest reliability, and good concurrent validity. A cut-off of 14 or above indicates the presence of clinically significant posttraumatic symptoms (Coffey, Gudmundsdottir, Beck, Palyo, & Miller, 2006).

The Beck Depression Inventory II (BDI II; Beck, Steer, & Brown, 1996) is a 21-item self-report measurement of the severity of depression; a score of 20 indicates moderate depression. The BDI-II has been found to have good test-retest reliability; high internal consistency; and acceptable content, construct, and criterion validity (Smarr & Keefer, 2011).

The Beck Anxiety Inventory (BAI; Beck & Steer, 1990) is a 21-item self-report instrument for assessing the severity of anxiety in adolescents and adults (Beck & Steer, 1990); a score of 17 indicates moderate anxiety. The BAI has been found to be highly internally consistent, with acceptable reliability (internal and test-retest) and convergent and discriminant validity (Fydrich, Dowdall, & Chambless, 1992).

Lotte completed the assessment at baseline, during treatment (NET session 4), after treatment, and at follow-up one month after the initial block of NET.

### 3.2. Course of treatment and assessment of progress

The treatment consisted of four sessions (45 minutes each) focused on establishing rapport, then 11 sessions of NET, and one booster session one month after the NET treatment. In the initial sessions, the client’s autobiography was incoherent and inconsistent. She was actively involved in goal setting and planning the course of therapy. In the NET sessions, one session (45 minutes) focused on psychoeducation and one session (60 minutes) was used to construct the lifeline using a ball of yarn, with rocks and flowers symbolizing significant events. The focus was not on specific trauma-details, but rather headlines such as ‘first sexual abuse’ or ‘Summer 1955’. Nine sessions (90–120 minutes) were focused on carrying out the narrative exposure according to the following stages: childhood, pretrauma (brief), first traumatic incident (detailed), posttrauma (brief), lifetime in between (very condensed), and second and following traumatic incidents (detailed), until all traumas were processed. The narrative exposure targeted the following: two specific incidents of sexual abuse at age 6 and age 10, a visit to the general physician at age 12 where he revealed the sexual abuse to her parents, the death of her brother when Lotte was age 15, suicide attempt at age 17, the infidelity at age 40, and the death of her ex-husband when she was 43. Some of these traumatic incidents occurred within the context of ongoing traumatic exposure. It was Lotte that chose these incidents, or ‘hot spots,’ as targets for therapy, based on the instructions to use rocks as symbols for significant events during the lifeline exercise. Re-reading of the written narrative marked the end of therapy.

### 3.3. Case conceptualization

Based on the initial assessment of PTSD symptoms and traumatic events, Lotte was presented with different treatment choices, including CBT and prolonged exposure therapy, but she chose to receive NET because of her preference for the chronological narrative approach targeting the traumas. The therapist conceptualized Lotte’s symptoms and complaints based on the theoretical foundation of NET (Schauer et al., 2011). She hypothesized that the explicit emphasis on chronology and Lotte’s life as a whole would be beneficial, based on Lotte’s experience of a neglectful upbringing, early beliefs of herself as unlovable, and deep-rooted feelings of shame and guilt as vulnerability factors. These feelings and beliefs were put in context of the Lotte’s life story in NET, in addition to exposure to her avoidance of various triggers related to her traumatic past.

## 4. Results

**Figure 1.**
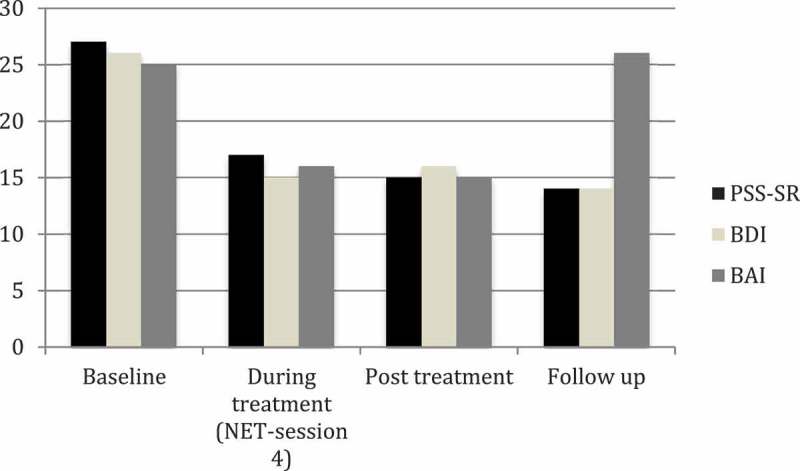
Measurement of symptoms of PTSD, depression, and anxiety during the course of treatment. PSS-SR = Posttraumatic Symptom Scale Self-Report, BDI = Beck Depression Inventory II, BAI = Beck Anxiety Inventory. Cut-offs for clinically significant levels of symptoms are 20 for the BDI-II, 17 for the BAI, and 14 for the PSS-SR.

At baseline, all clinical symptoms were at the clinically significant range, and there was a decline in posttraumatic, depressive, and anxiety symptoms by post-treatment (see Figure 1). Lotte described the lifeline exercise as a particularly helpful part of NET, and she reported her experience of improvement before treatment was terminated. At post-treatment, PTSD symptoms were near ‘mild’ levels, while both depressive and anxiety symptoms had decreased to the mild range, and there were no adverse events from the treatment. Lotte was not suicidal during treatment, had no symptoms of cognitive decline, and described good support from her current social network. At follow-up, Lotte’s gains in PTSD and depressive symptoms were maintained, while her anxiety symptoms returned to moderate levels.

## 5. Discussion

This case report demonstrated that NET could be administered safely and effectively to a 70-year-old woman with a complex trauma history and PTSD. She demonstrated improvements in PTSD and depressive symptoms, though her anxiety remained at a moderate level. The result is in line with preliminary studies indicating that life-review techniques and exposure-based psychotherapies might be beneficial for traumatized older adults (Böttche et al., 2012). The opinions concerning imaginal exposure in older adults has been mixed (Böttche et al., 2012), and this case report illustrates that it was well tolerated by this older adult subject. Results also align with the case report by Russo et al. (2001), demonstrating the efficacy of imaginal exposure in treating PTSD as a result of childhood sexual abuse in a 57-year-old woman. However, Russo et al. (2001) describes that the client received 60 sessions of therapy in total (including imaginal exposure and follow-up), while our client received 16 sessions (including narrative exposure and follow-up). In the Bichescu et al. (2007) report, comparing the efficacy of NET versus psychoeducation in Romanian older adult trauma survivors, they found that five NET sessions provided significant relief of PTSD and depressive symptoms, though four of the nine participants still met the criteria for PTSD at follow-up six months after treatment.

Strengths of the current study included repeated measures with strong psychometrics, an intervention with a solid theoretical foundation and strong empirical support, and a thorough client history that shows her diverse experiences. Her improvements did not appear to be due to her antidepressant medication, given that she had taken it for decades prior to and during this NET trial (and had substantial PTSD severity scores before the NET trial).

Treatment was not randomized. The client chose the treatment, and this self-selection may have enhanced the improvement in symptoms. The apparent effect of NET illustrated here could be explained by nonspecific therapy factors, such as therapist warmth or general therapeutic alliance. There is no longer-term follow-up (e.g. 6–12 months after the final therapy session); whether the treatment gains were lasting is not known. It is uncertain whether other populations of older patients (e.g. men, other racial or ethnic groups) with childhood and adult traumas will benefit from this approach. Anxiety symptoms decreased by post-treatment, but returned to pre-treatment levels at follow-up. That could indicate that the impact of treatment for those symptoms was temporary, or that the client would benefit from additional treatment.

## 6. Conclusions

This case study illustrates how NET was effective in treating PTSD and depressive symptoms in a Caucasian older Norwegian female with multiple childhood and lifetime traumatic experiences. In order to establish whether NET could be an evidence-based treatment for older adults with histories of multiple traumas, there is a need for controlled clinical trials. Based on this case study, such studies may be warranted.
